# Cooperation of RNA-Binding Proteins – a Focus on Roquin Function in T Cells

**DOI:** 10.3389/fimmu.2022.839762

**Published:** 2022-02-18

**Authors:** Gesine Behrens, Vigo Heissmeyer

**Affiliations:** ^1^ Institute for Immunology, Biomedical Center (BMC), Faculty of Medicine, Ludwig-Maximilians-Universität in Munich, Planegg-Martinsried, Germany; ^2^ Research Unit Molecular Immune Regulation, Helmholtz Zentrum München, Munich, Germany

**Keywords:** RNA-binding proteins, Roquin, Regnase-1, post-transcriptional gene regulation, cooperativity, autoimmunity, tumor immunity

## Abstract

Post-transcriptional gene regulation by RNA-binding proteins (RBPs) is important in the prevention of inflammatory and autoimmune diseases. With respect to T cell activation and differentiation, the RBPs Roquin-1/2 and Regnase-1 play pivotal roles by inducing degradation and/or translational silencing of target mRNAs. These targets encode important proinflammatory mediators and thus Roquin and Regnase-1 functions dampen cellular programs that can lead to inflammation and autoimmune disease. Recent findings demonstrate direct physical interaction of both RBPs. Here, we propose that cooperativity of *trans*-acting factors may be more generally used to reinforce the regulatory impact on selected targets and promote specific cell fate decisions. We develop this concept for Roquin and Regnase-1 function in resting and activated T cells and discuss the involvement in autoimmunity as well as how the therapeutic potential can be used in anti-tumor therapies.

## Introduction

In response to infections, our immune system first involves innate and then adaptive immune cells to clear pathogens. Lymphocytes recognize foreign structures derived from pathogens through their antigen receptors. One main purpose of antigen receptor signal transduction is to elicit specific changes in gene expression, which turn on selective differentiation programs. This is, for example, true for mature T cells recognizing antigen on antigen-presenting cells (APCs) in secondary lymphoid organs. As part of the adaptive immune response, T cells reprogram their metabolism, enter and progress in the cell cycle and commit to differentiation programs that lead to specific effector or memory functions. T cell receptor (TCR) signal transduction causes epigenetic changes and induces *de novo* transcription of mRNAs, whose expression can subsequently be controlled by numerous post-transcriptional regulatory mechanisms (1, 2). Transcription factors typically recognize DNA *cis*-elements in promoter regions of coding genes and recruit RNA polymerase II to initiate transcription from downstream transcription start sites. Similar to transcription factors recognizing DNA *cis*-elements of individual or composite binding sites, RNA-binding proteins (RBPs) are *trans*-acting factors recognizing *cis*-elements, which are mainly localized in 5’ or 3’-untranslated regions (UTR) of mRNAs, but can also be found in introns or in coding sequences. Again, such *cis*-element can be composed of binding sites for one or even several RBPs to function as a regulatory unit. The site-specific recognition then induces RBP-dependent types of post-transcriptional gene regulation. Prominent *cis*-elements for RBPs are adenylate-uridylate rich elements (AREs) or stem-loop (SL) structures (3–6). Besides RBPs, miRNAs are also important *trans*-acting factors involved in post-transcriptional gene regulation. miRNAs are a class of short (~22 nt) non-coding RNAs that, together with Argonaute (Ago) proteins, form the so-called miRISC complex, which recognizes sequence-specific sites in the 3´-UTR of their target mRNAs *via* base-pairing (7). Post-transcriptional regulation can affect nuclear pre-mRNAs and regulate processing, modification and export of mRNAs. On mature mRNAs in the cytoplasm, post-transcriptional regulation can have stabilizing effects or induce degradation as well as enhance or inhibit protein translation (8, 9). Dysregulation of gene expression in lymphocytes can cause inappropriate immune responses and lead to the development of autoimmunity or immunodeficiencies (10).

## Cooperativity of Transcription Factors in Response to T Cell Activation

Decades of research focusing on the regulation of transcription in T lymphocytes have uncovered a high degree of cooperation between transcription factors. For example, induced transcription of the gene encoding the cytokine interleukin (IL)-2 requires TCR engagement and costimulation, since the *Il2* gene contains a composite *cis*-element in the promoter. This *cis*-element requires the coinciding binding of NFAT, AP-1 and NF-κB, which are induced only during productive activation of T cells through both signals (11, 12). In fact, T cells can become anergic or exhausted if TCR stimulation triggers NFAT-dependent gene expression programs in the absence of AP-1 (13–16). In T cells, a physical interaction on composite DNA *cis*-elements has been observed for NFAT and AP-1 during productive T cell activation (17). On the other hand, alternative ternary complex formation of NFAT and Foxp3 was observed during Treg differentiation and function (18).

These well-investigated examples on the regulation of transcription illustrate how a limited set of *trans*-acting factors can, by engaging in a few different combinatorial activities that have been selected in evolution, allow for a number of fine-tuned, alternative and even opposing cell fate decisions.

## Cooperativity of *Trans*-Acting Factors Involved in Post-Transcriptional Gene Regulation

**Figure 1:**
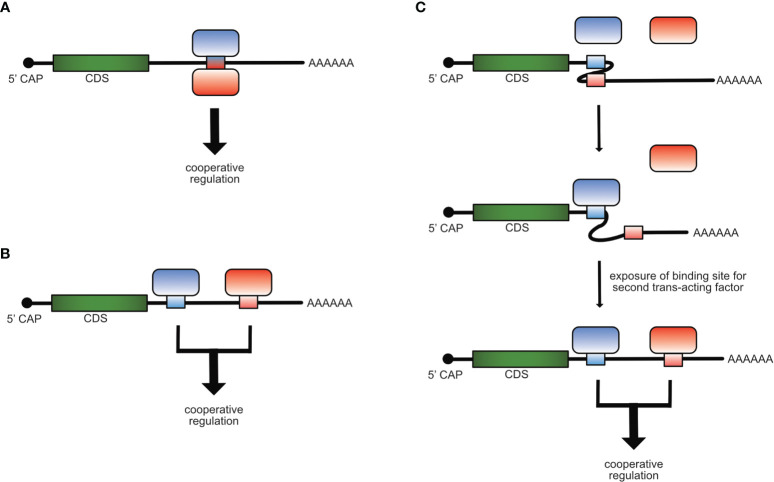
Mechanisms of cooperative post-transcriptional gene regulation of mRNAs. Cooperativity of RBPs can occur by binding of two trans-acting factors to the same *cis*-element **(A)**, to different *cis*-elements **(B)** on the same mRNA molecule or when the binding of one trans-acting factor induces a structural re-arrangement in the mRNA, which allows the access and binding of the second **(C)**.

Cooperativity in gene regulation can be observed when two or more factors function together and depend on each other to reach full regulatory impact. The main types of cooperativity in post-transcriptional gene regulation are: Physical cooperativity between different RBPs binding to the same *cis*-element where either both RBPs bind to the RNA of the *cis*-element at the same or at different binding sites or by forming a complex in which just one RBP binds directly to the RNA (**Figure 1A**), functional cooperativity by binding to different *cis*-elements on the same mRNA molecule (**Figure 1B**), or cooperativity due to changes in binding site accessibility, meaning that one *trans*-acting factor induces a redistribution within different conformational states of an RNA, thereby facilitating the access to binding sites for other *trans*-acting factors (**Figure 1C**) (19–25).

In the past, studies in the field of post-transcriptional gene regulation typically focused on the monocausal regulation performed by a single *trans*-acting factor recognizing a defined *cis*-element. However, based on the circumstantial evidence listed below we propose that cooperativity is an important aspect of post-transcriptional gene regulation, in general and in T cells.

1. The mRNAs of key proteins involved in cell fate decisions (e.g. regulators of transcription and signal transduction) are often unstable, contain long 3’-UTRs with multiple binding sites for several different *trans*-acting factors and show regulation by overlapping sets of post-transcriptional regulators. In line with this, T cell activation results in expression of transcripts with shorter 3´-UTRs due to usage of upstream polyadenylation sites, pointing to activation-dependent regulation due to altered 3´-UTR binding sites (26–28).

2. The high number of approximately 1200 canonical RBPs harboring a defined RNA-binding domain (RBD) and non-canonical RBPs without defined RBDs in human or mouse primary CD4+ T cells suggests an unexplored complexity of post-transcriptional gene regulation (29).

3. A recent study in human cell lines estimated an average of 22,000 3’UTR-located binding sites for each RBP (22), supporting the idea that most 3’ UTRs provide binding sites for several RBPs and/or miRNAs. The inducible T cell co-stimulator (ICOS), which encodes a costimulatory receptor that is essential for T cell help to B cells during the germinal center reaction, is a good example for a transcript regulated by different *trans*-acting factors. It responded to regulation by Roquin, Regnase-1, miRNAs and Wtap/m6A during the activation of murine T cells (29, 30).

Further evidence for the importance of cooperative post-transcriptional gene regulation in T cells comes from miRNA studies describing that several miRNAs which bind simultaneously to the same target mRNA molecule exerted stronger repression of the target mRNA than independent actions of each miRNA. In T cells, such functional cooperativity of miR-99a and miR-150 was involved in the repression of the mTOR mRNA promoting the conversion into iTreg cells (31).

Moreover, several publications have involved the Roquin-1 protein in physical or functional interactions with other post-transcriptional regulators of *ICOS* mRNA (32–34). Roquin was proposed to engage in physical interactions with Ago2 and miR-146a and thereby enable profound regulation of ICOS (33). A physical interaction of Roquin-1 with Nufip2 was identified in a siRNA screen for Roquin-1 cofactors and Nufip2 was shown to strengthen RNA-binding of Roquin-1 to tandem SL structures *in vitro* (32). More recently, overexpression of Celf1 or Igf2bp3 proteins were suggested to counteract Roquin-mediated repression of ICOS (29). Although these examples suggest intriguing cooperations, it is still unclear how the reported effects can be explained mechanistically. Moreover, the proposed interactions have not been substantiated by genetic proof *in vivo*.

In this review, we will focus on the RBP Roquin-1 and its interaction with Regnase-1, in which we showcase the importance of cooperativity of RBPs in T cells.

## Similarities in Roquin-1 and Regnase-1 Functions

Several recent papers have addressed a potential functional interdependence of Roquin and Regnase RBPs. Roquin-1, its redundantly-functioning paralog Roquin-2, as well as Regnase-1 are important regulators of T cell activation and differentiation (35–40). These proteins exhibit striking similarities in the following aspects:

1.) In addition to the ROQ RNA-binding domain (RBD) that specifies the binding of Roquin-1 and Roquin-2 to RNA (33, 41–44) and the PIN domain that enables Regnase-1 endoribonuclease function (45), all proteins harbor one CCCH zinc finger (46).

2.) Roquin-1, Roquin-2 and Regnase-1 are all cleaved by the paracaspase MALT1 in response to TCR stimulation (36, 38).

3.) Roquin-1, Roquin-2 and Regnase-1 have an overlapping set of mRNA targets including *Icos*, *cRel*, *Il6*, *Nfkbid* and also *Zc3h12a*, the Regnase-1 encoding mRNA (36, 38, 45, 47).

4.) Global mapping of binding sites of overexpressed Regnase-1 crosslinked to cellular mRNAs revealed comparable sequence determinants of Roquin-recognized SLs as defined by the constitutive decay element (CDE)  (3, 47).

5.) Roquin-1 and Regnase-1 have been found to induce post-transcriptional repression through rapid mRNA degradation as well as translational silencing (3, 38, 45, 48–50).

6.) Mice with T cell-specific deletions of the Roquin-1/2 encoding genes, *Rc3h1* and *Rc3h2* (DKO), or the Regnase-1 encoding gene *Zc3h12a* (KO) develop comparable phenotypes characterized by spontaneous activation of CD4^+^ and CD8^+^ T cells and accumulation of Tfh and GC B cells (30, 38, 40).

7.) Mice harboring a single point mutation in the *Rc3h1* gene that exchanges methionine 199 to arginine (M199R) in the Roquin-1 protein develop a severe systemic lupus erythematosus-like (SLE-like) phenotype, showing deregulation of the immune system and production of anti-nuclear antibodies (ANAs), a phenotype which can be also found in the Regnase-1 KO mouse (38, 39, 45).

This striking resemblance in Roquin-1 and Regnase-1 protein functions as well as mouse model phenotypes led to the hypothesis that these two proteins may have a cooperative function. Indeed, initial data with reporter assays using overexpressed RBPs showed impaired regulation of a CDE-containing TNF 3´-UTR fragment in the absence of one or the other, supporting the idea of cooperativity (36).

## Discrepancies and Challenges of Roquin and Regnase-1 Functional Interdependence

The proposed concept of cooperation (36, 51) has been questioned (46, 47), since Roquin and Regnase-1 RBPs also exhibit extensive differences:

On the mechanistic level, Roquin has been found to mediate degradation of its target mRNAs through the recruitment of the deadenylation machinery (3, 52) or through interactions with enhancers of decapping (50, 53). Regnase-1 harbors an intrinsic endonuclease activity, which requires Upf1 function and other factors involved in nonsense-mediated decay (NMD) (47, 54). Cellular localization studies revealed a close association of Regnase-1 with the ER and co-fractionation with translating ribosomes. Due to target expression kinetics, Regnase-1 was proposed to selectively regulate translationally active mRNAs in the early phase after LPS stimulation of fibroblasts. In contrast, Roquin localizes in P bodies and induces mRNA decay rather in fractions of polysome gradients that contain translationally inactive mRNAs and in the late phase of the innate immune response (47). These differences have led to the concept of an entirely compartmentalized function in which Roquin-1 and Regnase-1 regulate an overlapping set of target mRNAs *via* a common SL at different times, in different subcellular locations and through different mechanisms (46, 47).

A first approach of genetically combining the *sanroque* alleles with conditional ablation of Regnase-1 encoding alleles in T cells suggested non-redundant functions of both RBPs (55), however, this study did not discriminate T cell-intrinsic against known contributions of T cell-extrinsic functions of Roquin-1 (30, 56). The notion of more distinct functions was further supported by genetic inactivation of Regnase-1 in adoptively transferred tumor-antigen-specific cytotoxic T cells. In these experiments, Regnase-1 was shown to be a key regulator of T cell survival and metabolism. The inactivation of Regnase-1 encoding alleles by sgRNA/Cas9 targeting resulted in a profound improvement of anti-tumor responses (57, 58), while aspects of CD8^+^ T cell biology had not been studied for Roquin-1, yet.

## Cooperative Functions of Roquin and Regnase-1 in T Cells

Addressing the controversy about cooperativity or compartmentalization, the existence and importance of cooperative functions of Roquin-1 and Regnase-1 proteins in T cells has received strong support from our recent study, especially through reconstitution and complementation assays, the definition of structure/function-relationships and through the genetic disruption of physical interaction (30).

### Reconstitution and Functional Complementation

Reconstitution experiments overexpressing Roquin-1 or Regnase-1 in Roquin-1/2 DKO or Regnase-1 KO CD4^+^ T cells confirmed cooperative functions, since in the absence of Regnase-1, Roquin-1 showed a partial impairment in the ability to suppress ICOS expression and vice versa Regnase-1 overexpression did not downregulate ICOS in Roquin-deficient T cells (30). Also the regulation of endogenous Regnase-1 expression fully depended on cooperation, since ectopic Regnase-1 expression in Roquin-deficient T cells did not affect expression of *Zc3h12a* mRNA and Regnase-1 protein. Correspondingly, Regnase-1 protein was highly upregulated in Roquin-1/2 DKO T cells, reflecting that despite high endogenous Regnase-1 expression, the protein cannot complement for the loss of Roquin-1 and Roquin-2 function (30, 36). On the other hand, overexpression of other Regnase family members i.e. Regnase-2, Regnase-3 and Regnase-4 in Regnase-1 KO cells suppressed ICOS expression equally well and complemented for Regnase-1 loss-of-function. While the mRNAs encoding for ICOS and even more for Regnase-1 were cooperatively regulated, the *Tnfrsf4* mRNA encoding for Ox40 was not. Intriguingly, the MALT1-cleavage fragment of Roquin-1 (aa1-510), which was shown to be inactive in the regulation of the *Tnfrsf4* mRNA, retained a residual function to cooperatively repress *ICOS* or *Zc3h12a* mRNA in reconstitution experiments (3, 30, 36, 53).

### The Molecular Basis of Cooperation

A prerequisite for cooperativity is colocalization and proximity within the cell. Behrens et al., therefore confirmed colocalization of Roquin-1 and Regnase-1 in P bodies, verified their proximity *via* NanoBret assays and proved formation of a stable binary protein complex at a submicromolar affinity (KD=417nM) using Biacore measurements. Finally, a CDE-like SL (nt 194–212) of the *Zc3h12a* mRNA (59) was specifically bound by Roquin-1 and increasing Regnase-1 levels induced a supershift, indicating the formation of a ternary complex and cooperative binding of both RBPs on the same SL. Of note, whether Regnase-1 also binds to the RNA or only to Roquin-1 without directly contacting the RNA is not clear, yet. For interaction and cooperative target regulation with Regnase-1, the HEPN-ROQ domains of Roquin-1 were sufficient. The introduction of mutations on the surface of the ROQ domain defined the interaction surface, and intriguingly, the amino acid M199 was part of this binding site. Accordingly, mutations that impaired interaction of Roquin-1 and Regnase-1 also reduced the suppression of the cooperatively regulated targets *ICOS* and *Zc3h12a* in *in vitro* reconstitution experiments (30). However, a full mechanistic understanding how cooperative regulation of target mRNAs by Roquin and Regnase-1 is achieved, remains elusive and has to be part of future studies.

### Genetic Proof of Cooperative Regulation

The final proof of concept was achieved by the generation of mice harboring mutations, which were shown to interfere with Roquin-1 and Regnase-1 cooperation in *in vitro* studies. These mice developed a severe autoimmune phenotype with an increase in activated CD4^+^ and CD8^+^ T cells, accumulation of Tfh and GC B cells as well as the production of ANAs and showed a striking resemblance with *sanroque* mice (30). The interaction of Roquin and Regnase-1 in the repression of cooperative targets therefore proved to be essential for the prevention of autoimmune disease.

## Perspectives

The advantage of two trans-acting factors cooperating is to focus an enhanced regulatory impact on a defined set of targets. We think that in such a cooperation Roquin rather contributes the specificity of RNA-binding, while Regnase-1 may exert the strong post-transcriptional repression.

Cooperativity of two *trans*-acting factors depends on the respective expression levels and post-translational regulations as well as on the hierarchies of affinities and binding properties of interactions. Knowing and integrating these determinants may allow us to understand cooperative regulation of specific targets but not of others. From *in vitro* binding studies it appears that the RBD containing protein fragment of Roquin-1 has a higher affinity for CDE-like elements than the Regnase-1 fragment, whose binding was much more sensitive to nonspecific competitor RNA (30). An assessment of quantitative aspects in primary T cells and, considering heterogeneity, favorably on the single cell level, is challenging. However, these considerations can inspire novel approaches and help us to adjust the directions of future research. Future studies should for example focus on *cis*-element-encoded features, which define a cooperative target. Which other targets show cooperative regulation? By which post-transcriptional mechanism do Roquin and Regnase-1 suppress expression of their cooperatively regulated targets? Does it involve mechanisms of deadenylation or decapping which have been involved in Roquin-mediated target regulation (3, 50, 52), does it employ endonucleolytic cleavage, a main function of Regnase-1 (45)? Or can cooperative targets also be translationally silenced, a recently involved function that was ascribed to Roquin as well as Regnase-1 for certain targets (48, 49)? Does ternary complex formation of Roquin and Regnase-1 on RNA recruit effector molecules from individual or all of these mechanisms or even enable new interactions? It is likely that cooperative target regulation does not involve a prototypic Roquin-dependent post-transcriptional mechanism, since the amino-terminal MALT1 cleavage product of Roquin-1 (aa1-510) is sufficient to repress *Zc3h12a*/Regnase-1 and is also partially active to repress ICOS (30).

One intriguing aspect of Roquin/Regnase-1 cooperation is that, on the one hand, this mechanism controls T cell activity, and on the other hand, it is prominently involved in the repression of the *Zc3h12a* mRNA, which encodes the Regnase-1 protein itself. Thereby, this RBP interaction prevents autoimmunity but at the same time precisely adjusts Regnase-1 expression, which may also confer an evolutionary advantage potentially related to the toxicity that can be observed in overexpression studies of Regnase-1 (48, 60).

**Figure 2:**
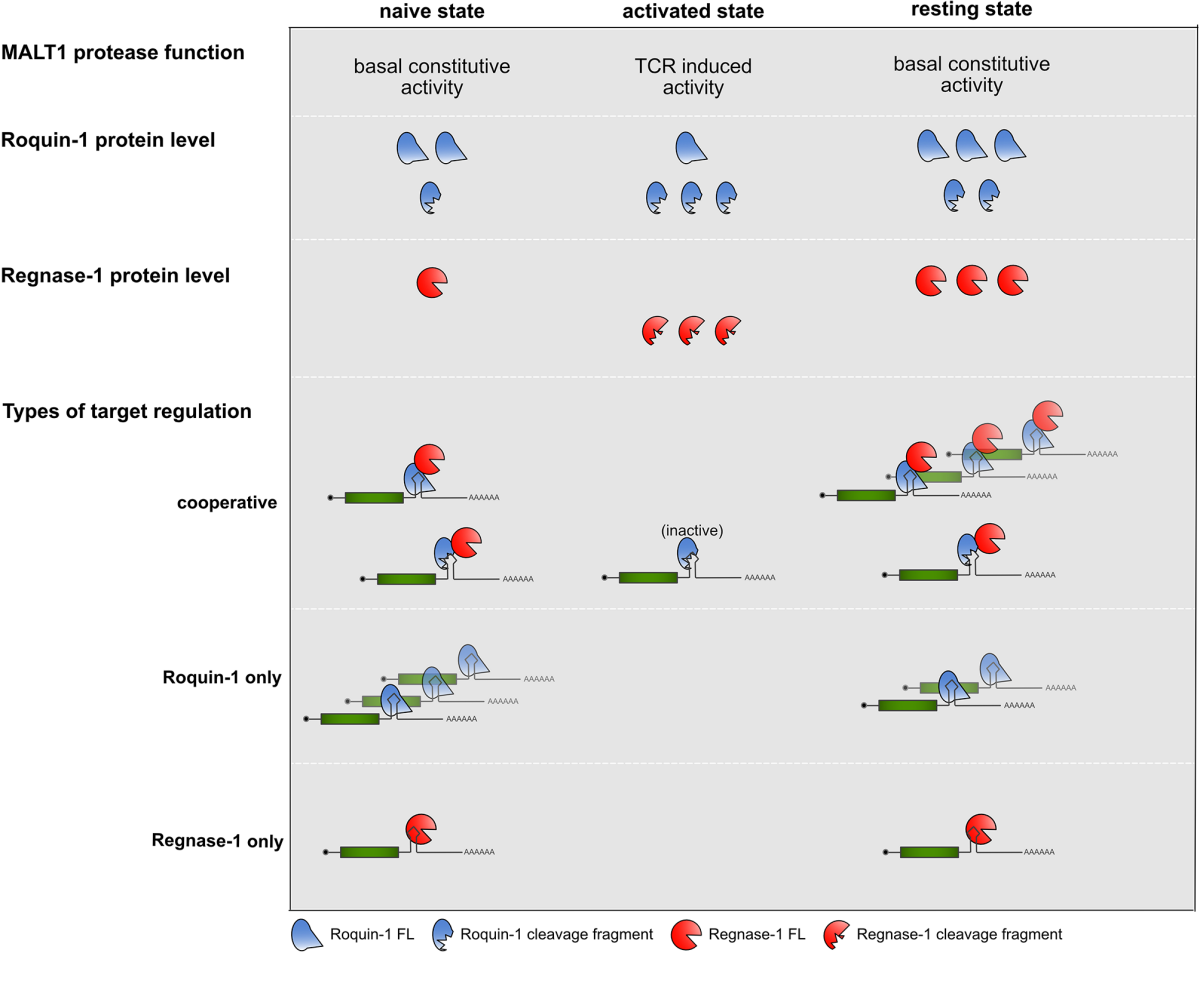
Summary of MALT1 protease function, Roquin-1 and Regnase-1 protein levels and types of target regulations in the different states of CD4^+^ T cells.

An important consideration is the kinetics of cooperation before, during and after TCR signal transduction (**Figure 2**). A recent report has now uncovered a previously unrecognized constitutive MALT1 protease activity, which is dampened through MALT1 interactions with TRAF6 through a so-far elusive mechanism. In this study the absence of TRAF6 or mutations in MALT1 impairing the interaction with TRAF6, enhanced the T cell activation independent protease activity of MALT1 and caused flagrant autoimmunity in mice (61). Reflecting constitutive MALT1 activity, Roquin-1 and Roquin-2 exhibit more constitutive cleavage by MALT1 than Regnase-1 in naive mature T cells (30). These findings lead to the question whether already naive T cells require Roquin cooperation with Regnase-1 to prevent autoimmunity. In line with this, Regnase-1 protein strongly increased prior to T cell activation, if Roquin function was genetically inactivated in naive T cells (30). Therefore, negative autoregulation of Regnase-1 levels as well as regulation of other cooperatively regulated targets are likely to occur prior to TCR-dependent activation of T cells, when Roquin expression levels are moderate and both, full-length as well as the truncated proteins are present, and only low levels of Regnase-1 full-length protein are expressed.

During TCR activation, when Roquin protein levels increase (30, 32), but are subject to MALT1 cleavage, no regulation of Roquin “only” targets occurs, for which the abundance of full-length Roquin is mandatory. In contrast, even in the absence of full-length Roquin the *Zc3h12a* mRNA could be suppressed by the Roquin-1 (aa1-510) MALT1 cleavage fragment when low amounts of induced and newly synthesized full-length Regnase-1 become available. This might reflect an important safe-guard mechanism which protects cells from overshooting Regnase-1 levels. Finally, upon removal of TCR signals, when Roquin and Regnase-1 re-appear in their full-length forms, they can switch off expression of cooperatively as well as Roquin and Regnase-1 independently regulated targets, efficiently stopping expression of pro-inflammatory mediators by several different modes of repression (30).

The *trans*-acting factors Roquin-1 and Regnase-1 were recently shown to be promising targets for therapeutic approaches. Despite experimental differences of either employing sgRNA-mediated inactivation of Regnase-1 or Roquin-1 or introducing point mutations, which disrupt the interaction of both RBPs, all studies report increased proliferation and persistence of the tumor-antigen-specific CD8^+^ T cells or CAR T cells in the tumor as well as efficient inhibition of tumor growth (30, 57, 58, 62). It is currently not clear whether these beneficial effects result from several different contributions from Roquin and Regnase-1 loss-of-function in addition to or only from cooperative regulation of targets. Importantly, inactivation of either Roquin-1 and Roquin-2 or Regnase-1 in T cells showed a similar enhancement but gradually different impacts on glycolysis and oxidative phosphorylation or on the proliferation and persistence of T cells as well as their effector functions (30). Moreover, the effect of Regnase-1 inactivation was specifically attributed to survival and proliferation, which depended on BATF expression (57). Consistent with such function, BATF3 was recently shown to prevent contraction in the pool-size of activated and differentiated effector CD8^+^ T cells as well as to be important for the development of memory (63). In contrast, gene editing to inactivate Roquin-1 boosted the proliferation of tumor-antigen-specific CD8^+^ T cells in a similar tumor setting, but this effect specifically required expression of the Roquin target IRF4 (36, 62). Determining the phenotype of tumor-antigen-specific CD8^+^ T cells with impaired Roquin-1/Regnase-1 interaction in the tumor we found that these cells showed greatly reduced expression of the exhaustion markers CD101, PD1 and Tox (30). Although, we have not yet identified or verified the cooperatively post-transcriptionally regulated targets that cause this phenotype, a very recent publication now presented an exciting connection of these findings: CAR T cells were shown to acquire enhanced functions in respect to expansion, persistence and memory formation through retroviral overexpression of BATF. Intriguingly, the physical interaction of BATF with the IRF4 transcription factor was essential for the improvement of anti-tumor responses and prevention of exhaustion in the tumor-specific CAR T cells (64). Together, these findings point at very promising new targets for improving immunotherapies. They also suggest cooperative gene regulation as a concept that can be exploited to achieve higher efficacy of adoptive T cell therapies, which is urgently needed to improve and expand treatment options for cancer patients.

## Author Contributions

All authors listed have made a substantial, direct, and intellectual contribution to the work, and approved it for publication.

## Funding

The work was supported by the German Research Foundation grants SPP 1935-273941853, SFB-TRR 338/12021-452881907 (project C02), SFB 1054-210592381 (project A03) as well as HE3359/7-1 (432656284) and HE3359/8-1 (444891219) as well as grants from the Wilhelm Sander (2018.082.2), Fritz Thyssen (Az.10.16.1.021.MN), Else Kröner-Fresenius (2015_A158) and Krebshilfe (70113538) Foundations.

## Conflict of Interest

The authors declare that the research was conducted in the absence of any commercial or financial relationships that could be construed as a potential conflict of interest.

## Publisher’s Note

All claims expressed in this article are solely those of the authors and do not necessarily represent those of their affiliated organizations, or those of the publisher, the editors and the reviewers. Any product that may be evaluated in this article, or claim that may be made by its manufacturer, is not guaranteed or endorsed by the publisher.
